# The impact of eHealth group interventions on the mental, behavioral, and physical health of adults: a systematic review protocol

**DOI:** 10.1186/s13643-020-01479-3

**Published:** 2020-09-23

**Authors:** Cheryl L. Currie, Richard Larouche, M. Lauren Voss, Erin K. Higa, Rae Spiwak, David Scott, Treena Tallow

**Affiliations:** 1 Faculty of Health Sciences, University of Lethbridge, 4401 University Dr, Lethbridge, AB T1K 3M4 Canada; 2 Manitoba Population Mental Health Research Group, Department of Psychiatry, Max Rady College of Medicine, University of Manitoba, Winnipeg, Canada; 3 University Library, University of Lethbridge, Lethbridge, Canada; 4 Alberta Health Services, Lethbridge, Canada

**Keywords:** Substance use, Mental health, eHealth, Group counseling, Systematic review, Physical activity

## Abstract

**Background:**

COVID-19 has resulted in an increased demand for eHealth services globally. There is emerging evidence for the efficacy for group eHealth interventions that support population-based mental health and wellbeing, but a systematic review is lacking. The primary objective of this systematic review is to summarize the evidence for eHealth group counseling and coaching programs for adults. A second objective is to assess, within studies selected for our primary objective, the impact of programs that encourage PA on outcomes compared to those that do not.

**Methods:**

Randomized controlled trials that assess the impact of eHealth group counseling or coaching programs on mental health, health behavior, or physical health activity among community-dwelling adults will be included. We will search the following electronic databases (from January 2005 onwards): MEDLINE, PsycINFO, CINHAL, and the Central Register of Controlled Trials. The primary outcomes will be changes in mental health conditions (e.g., depression, anxiety, stress, quality of life), behavioral health conditions (e.g., substance use, smoking, sexual behavior, eating behavior, medication adherence), and physical health conditions (e.g., coping with cancer, menopausal symptoms, arthritis pain). Secondary outcomes will be changes in physical activity. Two reviewers will independently screen all citations, full-text articles, and abstract data. Potential conflicts will be resolved through discussion with a third reviewer. A narrative synthesis without meta-analysis will be conducted. The strength of the body of evidence will be assessed using GRADE. The risk of bias in individual studies will be appraised using the Cochrane Risk of Bias 2.0 tool. Potential sources of gender bias in included studies will be considered at all stages of the planned review.

**Discussion:**

This review will contribute to the literature by providing evidence on the effectiveness of eHealth counseling and coaching programs delivered to adults in a group format.

**Systematic review registration:**

The protocol has been registered at the International Prospective Register of Systematic Reviews (PROSPERO: CRD42020187551).

## Background

The mental health and wellbeing of societies have been severely affected by the COVID-19 pandemic [1]. As outlined by the Secretary General of the United Nations, this problem is a priority that must be urgently addressed [1]. In Canada, approximately 25% of adults reported poor to fair mental health in April-May 2020 compared to 8% in 2018 [2]. Similar results have been reported in the UK, Italy, and Spain [3]. In China, almost half of all adults now report symptoms of anxiety and depression [4]. Individuals need help with social isolation, employment disruptions, financial distress, domestic violence, substance use, and grief and mourning for loved ones lost to COVID-19 [5]. Others, who are medically vulnerable to COVID-19 due to chronic health conditions, may be seeking resources and coaching so that they may become as healthy as possible in preparation for potential COVID-19 infection. As a result, the demand for mental health and health coaching services are increasing globally with COVID-19 spread [6]. If these services can be delivered effectively as eHealth interventions, they can address an important population health need while avoiding the face-to-face contact that fuels COVID-19 spread.

eHealth involves the use of information and communication technologies in the support of health and health-related activity [7]. eHealth interventions include services delivered online, by phone, and through mobile applications (“apps”). Systematic reviews suggest eHealth interventions can address mental, physical, and behavioral health concerns [8–13]. A problem is that positive outcomes are often tied to the intensity of therapist guidance, which has cost implications that can make the population scale-up of more effective interventions prohibitive [11]. A way to offset cost while maintaining the intensity of therapist guidance is to offer eHealth programs to *groups* of individuals rather than more standard one-on-one formats. While there is emerging evidence that eHealth interventions delivered in a group format can improve mental health and wellbeing, a systematic review is lacking [13–17].

The *primary objective* of this systematic review is to address a gap by summarizing evidence from randomized controlled trials that have assessed the impacts of *group* eHealth counseling and coaching programs among community-dwelling adults on acute or chronic mental health conditions or concerns, behavioral health concerns, or physical health concerns that they would like to address or cope with, relative to control or another form of intervention. In this review, we will define counseling and coaching broadly as the skilled use of a relationship to facilitate self-knowledge, wellness, and the optimal development of personal resources and resilience [18]. Given increased physical activity has been shown to improve mental and physical health generally, we will assess the impact of eHealth group counseling programs that encourage PA on these outcomes compared to those that do not as a secondary objective [19, 20].

## Methods

### Protocol and registration

This protocol has been registered within the International Prospective Register of Systematic Reviews (PROSPERO) database (registration number CRD42020187551) [21] and is being reported in accordance with the reporting guidance provided in the Preferred Reporting Items for Systematic Reviews and Meta-Analyses Protocols (PRISMA-P) statement [22, 23] (see checklist in Additional file 1). Any amendments to this protocol will be documented and published alongside the results of the systematic review.

### Eligibility criteria

#### Inclusion criteria

We will include randomized controlled trials, published in English or French, between January 2005 and onwards from any country. Only studies involving adult participants (18 years and older) living in community-based settings will be included. For example, adults living in care homes or prisons will be excluded. Pregnant women will be included. Studies selected for this review will assess group interventions delivered to adults who have acute or chronic mental health conditions or concerns (e.g., substance dependence, depression, psychological distress), behavioral health concerns (e.g., substance misuse), or physical health concerns (e.g., physical pain, menopausal symptoms) that they seek to address or cope with. This may include programs for health care workers, although this is not a requirement for inclusion.

Studies will be included if they have examined a group intervention that was entirely (100%) delivered in a live, synchronous, format with at least three sessions of guidance through video or phone conferencing, or group-based texting. In Table 1, we have outlined the types of eHealth delivery methods that will be included. Included studies will assess the effectiveness or comparative effectiveness of interventions. Studies that use one of the following comparators will be permitted: (i) standard care; (ii) control (wait-listed or no treatment); (iii) attention control group that includes some activities but differs from the intervention group in intensity, contacts, and/or time (e.g., providing a brochure for the topic under study) [24, 25]; (iv) attention placebo control that includes group activities that are similar to the intervention group in intensity, contacts, and/or time, but are missing a counseling or coaching component [26]; (v) unguided eHealth intervention; (vi) eHealth individual intervention; or (vii) non-eHealth group or individual in-person intervention.

**Table 1:** Types of interventions delivery

Intervention delivery	Included	Excluded
Videoconference	Participants can see and interact with each other in a synchronous way (e.g., skype)	Asynchronous, one-way messages, peer-led intervention, or one-on-one communication
Telephone conference	The intervention includes synchronous, live interaction between participants and facilitators	Asynchronous, one-way messages, peer-led intervention, or one-on-one communication
Text messages	Group text messaging	Asynchronous, one-way messages, peer-led intervention, or one-on-one communication
Mobile applications (apps)	The intervention app involves synchronous, live group communication	Asynchronous, one-way messages, peer-led intervention, or one-on-one communication
Virtual social networks (e.g., Facebook)	The intervention includes a synchronous group chat with live exchange between facilitator and participants	Asynchronous, one-way messages, peer-led intervention, or one-on-one communication
Websites and online communities	The intervention includes a synchronous group forum or chat with live exchange between facilitator and participants	Asynchronous, one-way messages, peer-led intervention, or one-on-one communication

Interventions must be led by a group leader who has training in mental health (e.g., a psychologist, psychiatrist, or counselor), health coaching, life coaching, or mindfulness/meditation. The group leader is required to have completed a certificate or a degree related to the intervention (e.g., health coaching certification, meditation certification, Master of Social Work degree).

#### Exclusion criteria

We will exclude non-randomized and observational studies. Studies published prior to 2005 will be excluded given the reduced availability of eHealth counseling and internet coverage in earlier years. Studies including participants under the age of 18 or those diagnosed with end-stage chronic disease or in palliative care will be excluded. Interventions delivered to adults living in institutionalized settings will be excluded (e.g., care homes, prison). Interventions that are not conducted entirely online, by phone, and/or group-based texting will be excluded. Peer-led groups and group leaders without a recognized certificate or degree related to the intervention will be excluded. For example, undergraduate students trained by the investigators to lead a mindfulness intervention would not meet inclusion criteria. However, undergraduate students who receive recognized certification in mindfulness teacher training will meet the inclusion criteria.

### Outcome measures

Studies that do not report at least one of the following outcomes will be excluded from the review.

#### Primary outcomes

The primary outcomes will be changes in acute or chronic mental health conditions or concerns, behavioral health concerns, or physical health conditions or concerns that have been examined using validated measures. Studies that examine differences in the incidence or prevalence of an outcome between intervention and comparator groups (e.g., number of unhealthy drinkers) as well as behaviors (e.g., number of alcoholic drinks per day) will be included. *Mental health outcomes or concerns* will include changes in outcomes such as depression, anxiety, stress and distress, substance dependence, well-being, quality of life, and other well-being constructs. *Behavioral health concerns* will include any health-related behavior that has been examined using a validated tool such as changes in substance use, smoking, sexual behavior, eating behavior, or medication adherence. *Physical health conditions or concerns* will include outcomes such as physiological changes such as improvements in blood pressure, as well as changes in symptom severity or perceived ability to cope with a physical illness (e.g., arthritic pain, menopausal symptoms, cancer). To be included in this review, adherence will have been examined using the mean number of modules/sessions completed and the percentage of persons that completed the whole treatment.

#### Secondary outcomes

Secondary outcomes will include changes in physical activity, whether or not it has been identified as a behavioral health concern by the participant, that has been measured using validated questionnaires or changes in device-measured activity patterns.

### Information sources and search strategy

The primary source of literature will be a structured search of major electronic databases (from January 2005 onwards): MEDLINE, PsycINFO, CINAHL, and also Cochrane Central Register of Controlled Trials (CENTRAL) in the Cochrane Library. The MEDLINE draft search strategy developed by a subject librarian (DS), and the primary investigator (CC) is provided in Additional file 2. This search strategy combines MeSH terms such as (mental health or mental disorders or behavioral symptoms) AND (Skype or Facetime or Zoom or Google+Hangouts) AND (counseling or psychotherapy or nursing or social work or yoga or meditation or mindfulness) were included in the search. In order to restrict our search to clinical trials, the search terms of (Randomized Controlled Trial or RCT* or randomi*) were also included.

### Data selection and screening process

Covidence will be used to manage records and data throughout the review [27]. Prior to screening, we will pilot test the screening instructions on five randomly selected studies to ensure consistency. If there is not a high percentage of agreement, we will further clarify the inclusion and exclusion criteria and re-test the process with five new studies. When 100% agreement is achieved, the team will start initial screening. Titles and abstracts will be independently screened by two reviewers (MLV, EH). In cases where a decision for exclusion or potential inclusion cannot be made by the title/abstract, the full text will be retrieved. Consensus meetings to reconcile disagreements will occur at each 30% interval of records screened. After initial screening, full-text copies of the articles will be obtained and independently reviewed by two authors (MLV, EH) to ensure inclusion criteria are met. If needed, consensus on the final inclusion will be achieved by discussing it with a third reviewer (CC or RL).

### Data extraction, evaluation, and synthesis

A data extraction form will be designed in covidence and used to extract equivalent information from each study. Data extraction forms will be piloted initially on a small number of included studies. Subsequently, each of the included studies will be abstracted by two reviewers, independently, and potential conflicts will be resolved through discussion. Authors of primary publications will be contacted (by email) for data clarifications or missing outcome data, as necessary. We will follow-up twice with each author, and wait 1 month after our initial email before excluding an article we are unclear about from the review. Extracted data will include funding sources, country, setting, author, RCT type (i.e., parallel, crossover), blinding and randomization methods, sample size calculation, inclusion and exclusion criteria, group baseline differences, baseline characteristics, intervention details (i.e., number of weeks, theoretical framework, frequency of sessions, length of sessions, expertise of facilitator, group size, incentives/honoraria), control group type, and outcome measurements. The Cochrane Handbook [28] and Synthesis Without Meta-Analysis (SWiM) [29] reporting guidelines will be used to conduct and report data synthesis, respectively. Due to the expected variation in interventions and reporting of outcomes, a meta-analysis will not be performed [30]. Instead, findings will be synthesized narratively and a summary will be presented in a table that includes setting, design, country, population, sample size, analytic method, relationships between group eHealth counseling or coaching and outcomes of interest, and relevant results. We will conduct a summary of findings for each outcome in which five or more studies are selected for review. For example, if five studies selected for the review examined anxiety as an outcome, we will do a summary of findings for that outcome. We have also planned a summary of findings by gender, and the intensity/duration of the interventions. These results will be presented in the “Summary of findings” tables created using GRADEpro [31]. The risk of bias will be assessed and reported on by MLV and EH using the Cochrane Risk of Bias tool [32]. Discrepancies will be resolved in a discussion with CC and RL. The risk of bias domains to be analyzed are (a) random sequence generation, (b) allocation concealment, (c) blinding, (d) incomplete outcome data, and (e) selective reporting and other bias. In psychological interventions, blinding is not possible resulting in a high risk of bias rating of (c), which we will discuss in our findings. Higher quality records will be prioritized when drawing conclusions. The role of sex and gender will be considered as well as possible gender biases at all stages of the review process from article selection and synthesis to knowledge mobilization, e.g., creation of gender-specific guidelines for eHealth therapies. Finally, we will narratively summarize the implications of findings as they may pertain to the documented impacts that COVID-19 has had on substance misuse, mental health, and well-being within adult populations to inform the decisions of governments, communities, and health care organizations responding to the pandemic.

### Confidence in cumulative evidence assessment

We will evaluate the strength of the body of cumulative evidence using the GRADE (Grading of Recommendations, Assessment, Development and Evaluation) [33] approach. We will assess the strength of evidence using five criteria: risk of bias, inconsistency, indirectness, imprecision, and publication bias. We will rate the overall level of certainty for each outcome as high, moderate, low, or very low. Results will be presented in tables for each primary outcome.

### Patient and public involvement

We have developed this study in collaboration with various stakeholders and knowledge users to ensure its applicability. In particular, we are working with indigenous stakeholders to ensure alignment with needs and priorities. There are concerns about the risk posed by COVID-19 for elders, given their centrality within many indigenous cultures, as well as the risk posed to adults with mental and physical health concerns, and a desire for eHealth programs that can strengthen resilience in the face of COVID-19. We seek to provide a systematic review that will be of maximum utility for indigenous organizations who are mobilizing to address this need in their communities.

## Discussion

This review will contribute to the literature by summarizing the evidence for eHealth counseling and coaching programs that can be delivered to populations in a group format and thus in a cost-effective way without losing the element of therapist interaction. This review may also contribute to our understanding of how physical activity may be encouraged within these interventions as a drug-free way to strengthen well-being. We expect individual studies may be limited by low intervention adherence/attendance, loss to follow-up post-intervention, and risk of bias due to the non-blinding that occurs in mental health and behavioral interventions. At a larger review level, we anticipate that some outcomes may not have been sufficiently studied, resulting in inconclusive review results. As part of our review, we will identify knowledge strengths and gaps related to this area of inquiry. The findings of this review will be shared through peer-reviewed publications in academic journals, conference presentations, and knowledge translation packages to inform community knowledge users and government decision-makers developing effective interventions to support the mental, behavioral, and physical well-being of the populations they serve.

## Supplementary information

**Additional file 1.**

**Additional file 2: Table S2.** Search Strategy.

## Data Availability

Not applicable.

## Abbreviations

CINHAL: Cumulative Index of Nursing and Allied Health Literature
COVID-19: Coronavirus disease 2019
GRADE: Grading of Recommendations, Assessment, Development and Evaluation
MeSH: Medical subject headings
PRISMA: Preferred Reporting Items for Systematic Reviews and Meta-Analysis
PRISMA-P: Preferred Reporting Items for Systematic Reviews and Meta-Analysis for Protocols
PROSPERO: Prospective Register of Systematic Reviews
SWiM: Synthesis without meta-analysis
