# Supplementary material for: The impact of eHealth group interventions on the mental, behavioral, and physical health of adults: a systematic review protocol
Source: Syst Rev. 2020 Sep 23;9:217. doi: 10.1186/s13643-020-01479-3 (PMC7513289; doi:10.1186/s13643-020-01479-3)
Supplement: Supplementary file 2 — Additional file 2: Table S2. Search Strategy. [file 13643_2020_1479_MOESM2_ESM.docx]

**Table 2**. Search Strategy

| 1. Mental Health/ [MeSH] 2. exp Mental Disorders/ [MeSH] 3. exp Behavioral Symptoms/ [MeSH] 4. (((mental* or psychological*) adj3 (health* or well* or disorder* or ill*)) or anxi* or depress* or neuros* or psychiatric or stress* or distress* or emotion* or aggress* or trauma* or suicid* or bereav* or grief or griev* or mourn* or addict* or alcoholism or ((substance* or drug* or alcohol*) adj3 (us* or misus* or abus* or dependen*))).mp. 5. or/1-4 6. exp Videoconferencing/ [MeSH] 7. exp Telemedicine/ {MeSH] 8. exp Internet/ {MeSH] or exp Telephone/ [MeSH] 9. (telehealth or telemedicine or ehealth or video*).mp. 10. (Skype or Facetime or Zoom or Google+Hangouts).mp. 11. (internet or web or online or telephon* or phone or phoning or phones or phoned or SMS or text messag* or texting or texted).mp. 12. (distance or remote).mp. 13. or/6-12 14. exp Counseling/ [MeSH] or exp Psychotherapy/ [MeSH] or exp Nursing/ [MeSH] or exp Social Work/ [MeSH] or Yoga/ [MeSH] or Meditation/ [MeSH] or Mindfulness/ [MeSH] 15. (counsel* or motivational interview* or coach* or psychotherap* or social work* or nurs* or kinesiolog* or yoga or meditat* or mindfulness).mp. 16. or/14-15 17. 13 and 16 18. Distance Counseling/ [MeSH] 19. (e-therap* or etherap* or e-counsel* or ecounsel* or telepsycholog* or "tele-mental health" or e-coach* or ecoach*).mp. 20. or/17-19 21. group*.mp. 22. exp Adult/ [MeSH] 23. adult*.mp. 24. or/22-23 25. exp Randomized Controlled Trial/ [MeSH] 26. (randomi* or randomly).mp, pt. 27. RCT*.mp 28. or/22-24 29. 5 and 20 and 21 and 24 and 28 30. limit 29 to (yr="2005 -Current" and (english or french)) |
| --- |
